# Multifocal stenosis in purulent appendicitis with fecalith

**DOI:** 10.1055/a-2239-3401

**Published:** 2024-02-02

**Authors:** Fan Wang, Yue Zhu, Qiu Zhao, Hongling Wang

**Affiliations:** 1 89674 Department of Gastroenterology, Zhongnan Hospital of Wuhan University, Wuhan, China; 2 Hubei Clinical Center and Key Lab of Intestinal and Colorectal Diseases, Wuhan, China

A 32-year-old woman was admitted for abdominal pain around the navel for over 1 year. At the local hospital, an abdominal CT scan showed appendicitis and an appendiceal fecalith. Subsequently, endoscopic retrograde appendicitis therapy was prepared but failed because the guidewire and catheter could not access the appendix lumen 1 . As a result, the patient was referred to our hospital for further treatment.

After admission, abdominal ultrasonography confirmed the appendiceal fecalith (0.82 × 0.29 cm; appendix size: 5.0 × 0.7 cm). Endoscopic retrograde appendicitis therapy using an appendoscope (eyeMAX, 9-Fr; Micro-Tech (Nanjing) Co., Ltd., Nanjing, China) was planned. During the operation, the appendoscope was inserted into the appendiceal lumen and detected apparent mucosal erosion and suppuration ( Video 1 , Fig. 1 ). Lumen stenosis was found in three sites. When it was difficult to distinguish the stenosis from the appendix terminus, a guidewire was used for exploration. Once the stenosis was determined, it was repeatedly dilated with the appendoscope body. Finally, we found the fecalith at the end of the appendix, removed the stone with a basket, and fully washed the cavity with 0.5% metronidazole ( Fig. 2 , Fig. 3 , Fig. 4 Fig. 5 ). The patient’s abdominal pain was relieved immediately after the procedure, and she was discharged 2 days later. No recurrence or any other adverse event was noted during a 2-month follow-up. To the best of our knowledge, this is the first reported endoscopic diagnosis and treatment of multifocal stenosis in purulent appendicitis with fecalith.

Endoscopic diagnosis and treatment of multifocal stenosis in purulent appendicitis with fecalith.Video 1

**Fig. 1:**
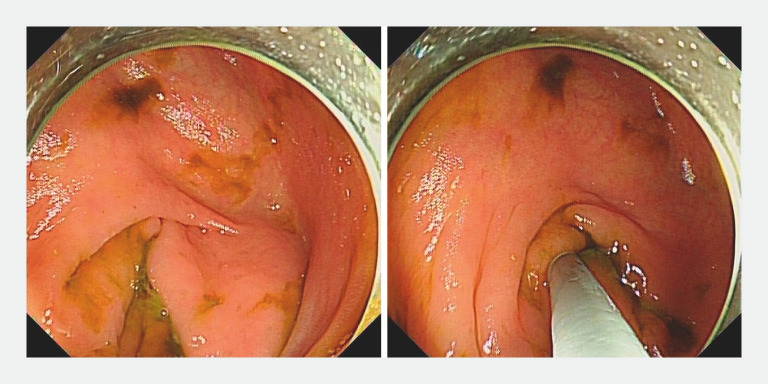
Appendoscope passing through the appendiceal orifice.

**Fig. 2:**
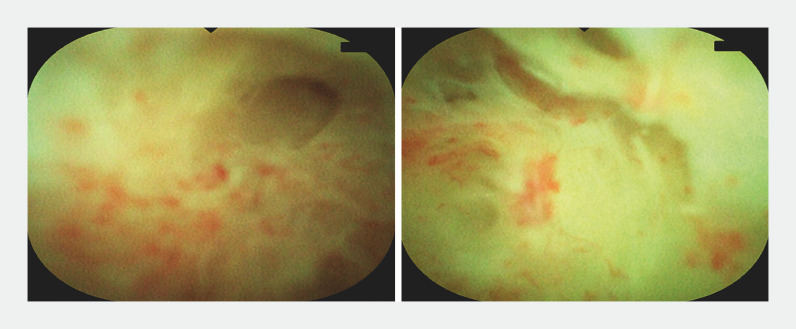
Passing through the first stenosis.

**Fig. 3:**
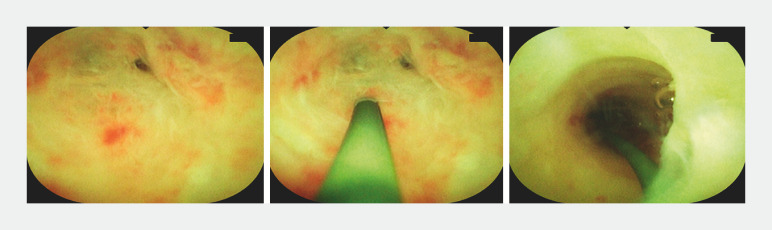
Passing through the second stenosis with the help of a guidewire.

**Fig. 4:**
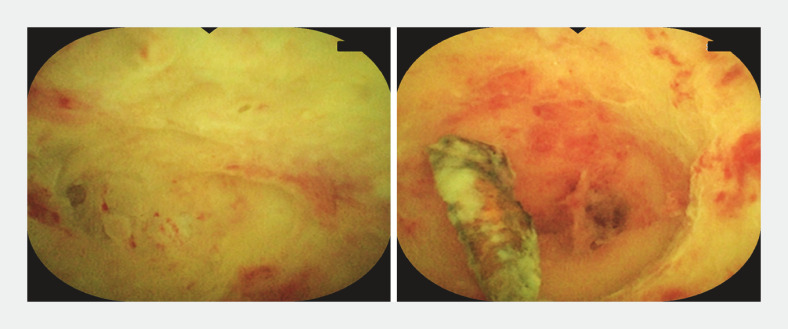
Passing through the third stenosis and detecting the fecalith.

**Fig. 5:**
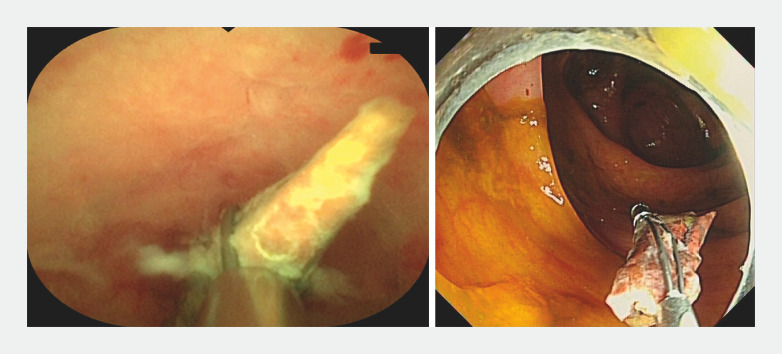
Removing the fecalith with a basket.

Endoscopy_UCTN_Code_TTT_1AQ_2AJ

## Notes

Endoscopy E-Videos https://eref.thieme.de/e-videos
*E-Videos* is an open access online section of the journal *Endoscopy* , reporting on interesting cases and new techniques in gastroenterological endoscopy. All papers include a high-quality video and are published with a Creative Commons CC-BY license. Endoscopy E-Videos qualify for HINARI discounts and waivers and eligibility is automatically checked during the submission process. We grant 100% waivers to articles whose corresponding authors are based in Group A countries and 50% waivers to those who are based in Group B countries as classified by Research4Life (see: https://www.research4life.org/access/eligibility/ ). This section has its own submission website at https://mc.manuscriptcentral.com/e-videos .
